# T6SS: The bacterial "fight club" in the host gut

**DOI:** 10.1371/journal.ppat.1006325

**Published:** 2017-06-08

**Authors:** Thibault G. Sana, Kyler A. Lugo, Denise M. Monack

**Affiliations:** Department of Microbiology and Immunology, Stanford University School of Medicine, Stanford, California, United States of America; Geisel School of Medicine at Dartmouth, UNITED STATES

## Introduction

The mammalian gut is home to a densely populated community of microorganisms that not only provide their host with nutritional benefits but also offer protection against foreign pathogens [1]. Since the gut is an environment limited in both space and nutrients, these microbes have evolved multiple mechanisms and strategies to either coexist or compete with other organisms that share the same resources. While some species will switch their metabolism to utilize secondary nutrients, others opt to take a more direct approach and directly kill their competitors by releasing chemical compounds or by secreting effectors via specific secretion systems [2] such as the type VI secretion system (T6SS).

T6SSs are contact-dependent secretion machineries capable of directly injecting toxins into other bacteria as well as eukaryotic cells [3]. Recent studies have highlighted the role of T6SS-dependent antibacterial responses in interbacterial competition in the mammalian gut [4–8], suggesting that T6SSs may be important in not only shaping microbial community composition but also governing interactions between the microbiota and invading pathogens. In this short article, we discuss recent advances in our understanding of how commensal intestinal microbiota and enteric bacterial pathogens use T6SS-mediated antibacterial activity to influence host health and whether manipulating the T6SS could be used for potential therapies in the future.

## What is the T6SS and how does it function?

To manipulate and control their local environment, bacteria often secrete proteins and effectors into the surrounding extracellular medium or directly into target cells using complex nanomachines called secretion systems. While these systems can vastly differ in function and composition, the T6SS is structurally homologous to a contractile T4 bacteriophage tail [9] and shares many evolutionarily conserved core components found in the T4 bacteriophages [3].

In regard to its mechanism of action, the T6SS is sometimes compared to a crossbow or speargun in that contractile sheath proteins ClpV-interacting protein A and B (VipA-VipB) cover a nanotube of stacked hexamers (hemolysin-coregulated proteins [Hcp]) that make up the body of the arrow [10]. Once the sheath contracts, the nanotube, which is often loaded with effectors, is injected into the target cell [11]. After firing, the sheath is recycled by the ATPase caseinolytic peptidase V (ClpV) [12], effectively resetting the system so that a new arrow could be fired again.

T6SSs are well-conserved amongst gram-negative bacteria, especially throughout the Proteobacteria and Bacteroidetes phyla [13]. Although the first data describing secreted components of T6SS (Hcp’s) can be found as early as 1996 [14], the secretion system was named T6SS by John Mekalanos’s group in 2006 (for a review, see [15]) and was initially thought to simply target eukaryotic cells. Since 2010, several studies found these machineries could also serve as an antibacterial weapon, greatly expanding our understanding of the role and function of the T6SS (for a review, see [16]). Some bacteria use their T6SS for both antiprokaryotic and anti-eukaryotic functions. For example, *Pseudomonas aeruginosa* encodes 3 distinct T6SS loci in its genome, H1- to H3-T6SS, that allow the pathogen to target both eukaryotic and prokaryotic cells. While H1-T6SS is specifically an antibacterial weapon, H2- and H3-T6SS can target both bacterial and eukaryotic cells and mediate invasion of nonphagocytic cells (for a review, see [17]). T6SS-encoding bacteria can mount a counterattack by expressing antitoxins that bind T6SS-injected toxins to inactivate them [18].

## Why is it relevant to study the role of T6SS in the gut?

Once it was established that the T6SS can serve as an antibacterial weapon, researchers wondered whether this activity is important in modulating bacterial interactions in the mammalian gut. By studying the gut commensal bacterium *Bacteroides fragilis*, Wexler and colleagues determined that more than 10^9^ T6SS-firing events occur per minute per gram of colonic contents, and that these microbial symbionts require their T6SS to persist in the gut [4]. Moreover, 130 T6SS loci were identified within the 205 human Bacteroidales genomes analyzed, suggesting that about a quarter of the human gut microbiota encode at least 1 T6SS [19] and that T6SS genetic elements may be transferable between Bacteroidales species [20]. Based on the dramatic firing rate and the large distribution of such machineries amongst gut commensals’ genomes, it is reasonable to hypothesize that T6SSs are key players involved in modulating ecological dynamics of the gut microbiota.

**Fig 1:**
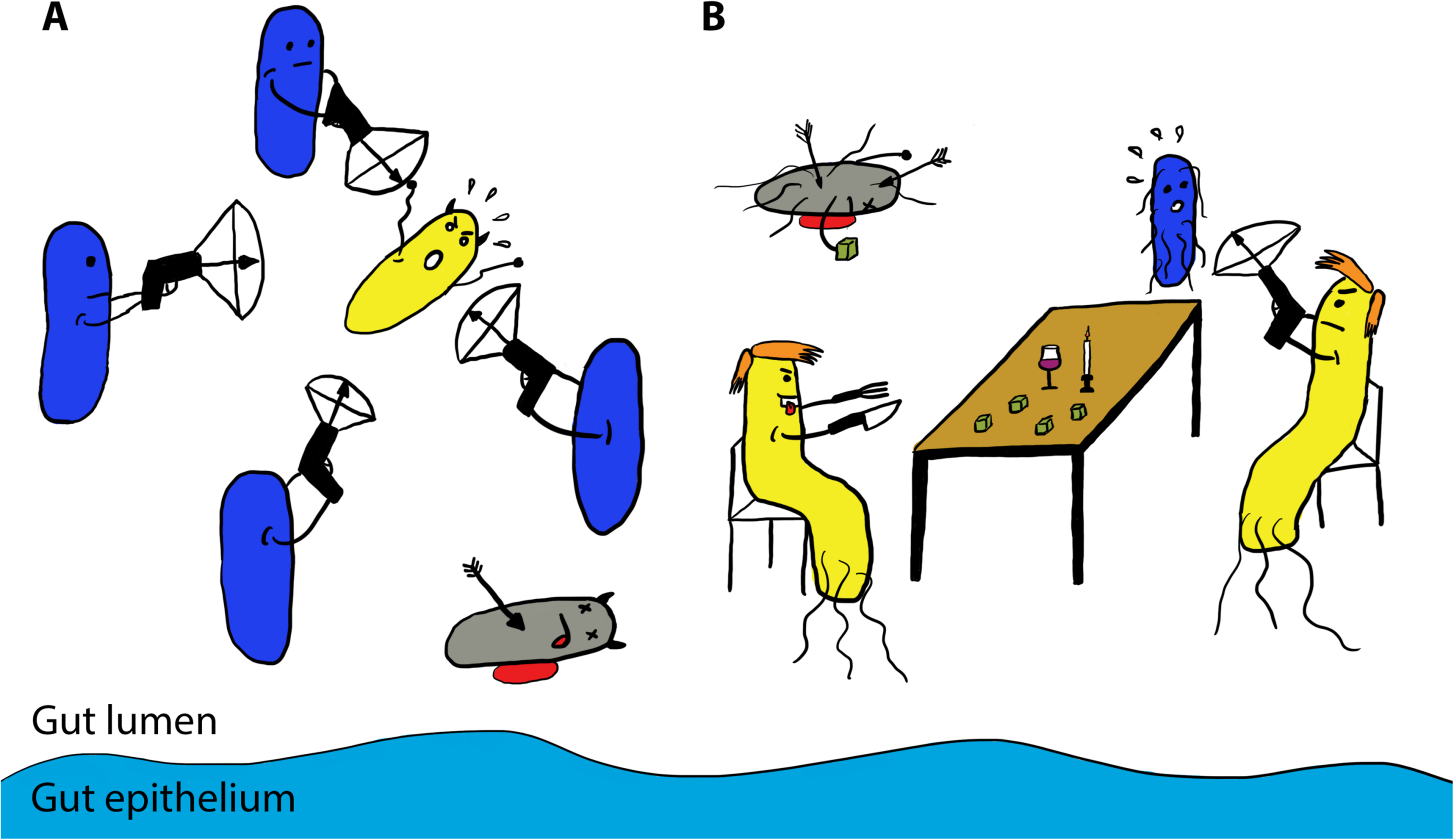
The T6SS-mediated bacterial warfare in the host gut. **(A)** Commensal *Bacteriodes fragilis* bacteria (in blue) target and kill enterotoxigenic *B*. *fragilis* (in yellow) in a T6SS-dependent manner (shown as crossbows), providing colonization resistance to the host. **(B)** *Salmonella* Typhimurium (in yellow) uses its T6SS crossbow to kill *Klebsiella oxytoca* (in blue), a potential nutritional competitor, allowing *Salmonella* to expand in the host gut. Dead bacteria are represented in grey, the gut epithelium is represented in light blue, and green cubes represent similar sugars metabolized by *Salmonella and Klebsiella*.

That said, the rules that govern the T6SS-dependent warfare in the mammalian gut are complicated and are still being worked out. One recent publication utilized in vitro killing assays to demonstrate that *B*. *fragilis* uses its T6SS to antagonize numerous Bacteroidales species isolated from the human gut [8]. In addition to killing other commensal bacteria, nontoxigenic *B*. *fragilis* limits acquisition of pathogenic enterotoxigenic *B*. *fragilis* in the guts of mice [5] (Fig 1A). This strain-specific competition with toxigenic *B*. *fragilis* was dependent on T6SS and the presence of a specific effector-immunity pair [5]. Altogether, T6SSs in commensal bacteria play an important role in defense against invading pathogens and might be one major player dictating microbial composition in the host gut, therefore greatly influencing host health.

## Are T6SSs utilized by enteric pathogens in the gut?

T6SSs are not limited to commensal bacteria. Many gram-negative enteric pathogens, including *Vibrio cholerae*, *Campylobacter jejuni*, *Shigella flexneri*, and *Citrobacter rodentium*, contain T6SSs. Moreover, both *V*. *cholerae* and *C*. *rodentium* utilize their T6SS to kill other bacteria in vitro [21–22]. In fact, in vitro studies have shown that the *V*. *cholerae* T6SS is activated by mucins and by microbiota-modified bile salt [23]. Consistent with these findings, an intact T6SS is required for *V*. *cholerae* colonization of the guts of infant rabbits [24].

To determine whether a T6SS-dependent anticommensal activity is required for the pathogen to effectively colonize and proliferate in the host gut, we recently used a *Salmonella enterica* serovar Typhimurium mouse model and found *S*. Typhimurium kills a commensal bacterium *Klebsiella oxytoca* in vitro and in the host gut in a T6SS-dependent manner [7]. This killing was magnified in the presence of bile salts in vitro and required the T6SS-dependent antibacterial toxin type VI amidase effector (Tae4). While it is still too early to fully understand why *Salmonella* might target *K*. *oxytoca*, recent studies have found *Klebsiella* is capable of metabolizing sugars that are similar to the sugars utilized by *Salmonella* in the guts of mice [25], suggesting that the pathogen might be killing a nutritional competitor through its T6SS crossbow (Fig 1B).

The presence of T6SS loci in many other gram-negative enteric pathogens supports the hypothesis that T6SS may be an evolutionarily conserved mechanism used by gram-negative enteric pathogens to establish themselves in the heavily populated gut to further cause disease.

## Is there any potential biomedical application of this knowledge?

The answer is “possibly.” Though there is much to be discovered, it may be possible to engineer probiotic commensal species that produce and use T6SSs that are designed to specifically kill certain enteric pathogens. Engineering such a commensal may come at a cost, however, depending on how specific the T6SS-dependent antibacterial response is. If the T6SS’s target isn’t specific enough, it may also kill resident gut bacteria that are important for maintaining homeostasis and thus negatively impact the health of the host.

**Fig 2:**
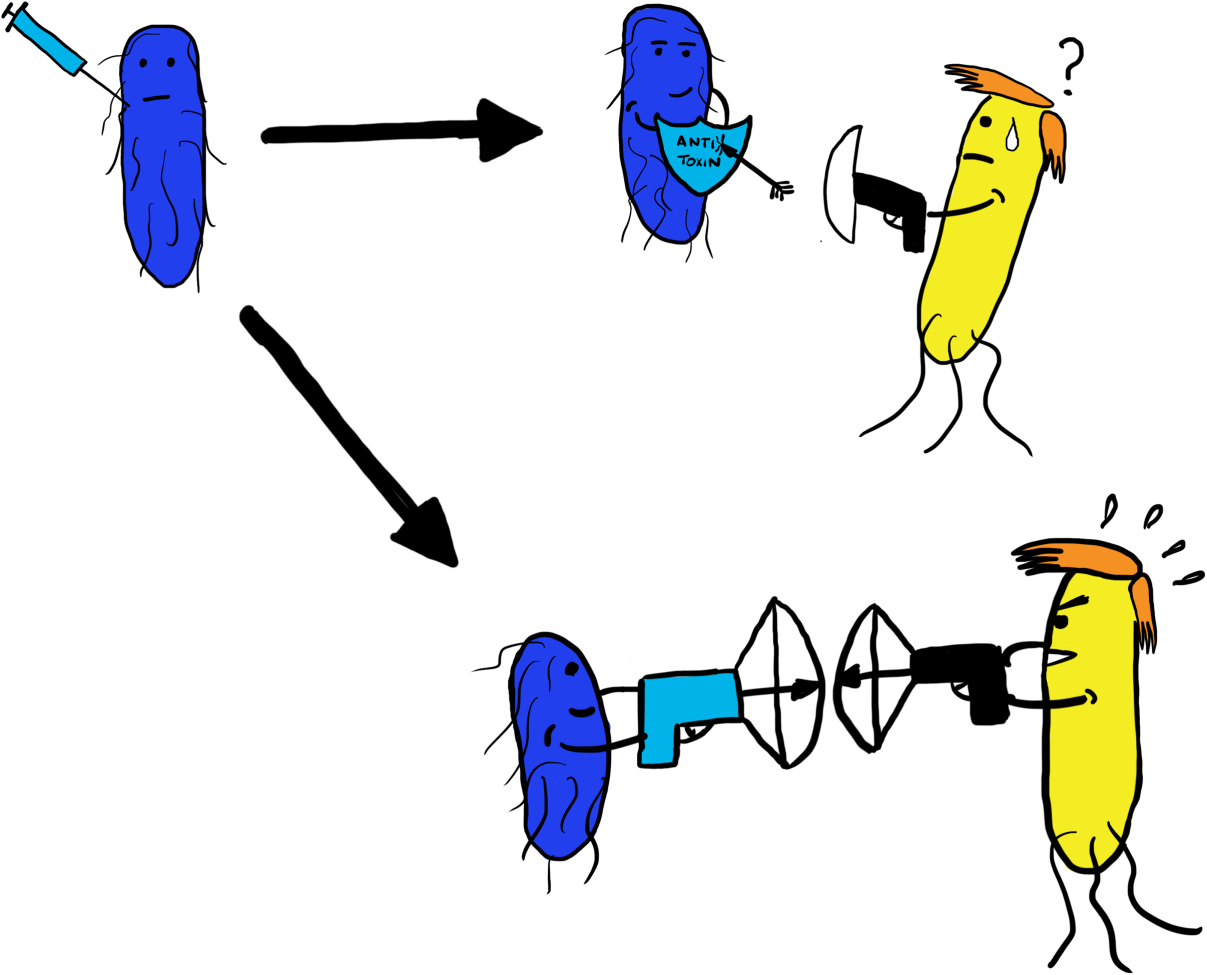
Engineering probiotic bacteria to fight enteric pathogens? Commensal bacteria (in blue) could be genetically engineered (depicted with a syringe) to be resistant to a pathogen’s T6SS attack by providing them with genes encoding an antitoxin (depicted as a light blue shield) or by providing them with their own T6SS (shown as a light blue crossbow) that specifically targets the pathogen (in yellow).

Alternatively, if we understand which specific commensals are targeted by a pathogen, it might be possible to provide them with the appropriate antitoxin genes to prevent them from being killed. This engineered commensal would therefore become immune to the pathogen’s T6SS attack and become a direct competitor for the pathogen, thus providing the host with colonization resistance to the pathogen (Fig 2).

## Is there a secret bacterial warfare in the gut?

In a way, yes, there is. It seems bacteria are secretly fighting amongst one another in the gut via their T6SSs. While much is yet to be discovered, T6SSs provide their host with protection against invading pathogens and may be responsible for dictating resident microbiota compositions. On the other hand, pathogens have figured out how to breach this defense mechanism, leading to their successful colonization of the mammalian gut.

Based on our current knowledge, the bacterial warfare in the gut does not appear to cause adverse effects such as inflammation in the gut, keeping this war a secret from the host. Maybe it’s because microbes don’t want us to know, since the first rule of Fight Club is “You do not talk about Fight Club.”
